# Liver segmental volumes and their relationship with 5-year prognostication

**DOI:** 10.1007/s00261-024-04552-w

**Published:** 2024-09-10

**Authors:** Damiano Catucci, Joris Hrycyk, Naomi Franziska Lange, Verena Carola Obmann, Annalisa Berzigotti, Michael Patrick Brönnimann, Lukas Zbinden, Kady Fischer, Dominik Paul Guensch, Lukas Ebner, Justus Roos, Andreas Christe, Adrian Thomas Huber

**Affiliations:** 1 Department of Diagnostic, Interventional and Pediatric Radiology, Inselspital, Bern University Hospital, University of Bern, Freiburgstrasse 10, 3010 Bern, Switzerland; 2 Graduate School for Health Sciences, University of Bern, Bern, Switzerland; 3 Institute for Diagnostic and Interventional Neuroradiology, Inselspital, Bern University Hospital, University of Bern, Bern, Switzerland; 4 Hepatology, Department of Visceral Surgery and Medicine, Inselspital, Bern University Hospital, University of Bern, Bern, Switzerland; 5 ARTORG Center for Biomedical Engineering, Bern, Switzerland; 6 Anesthesiology Department, Inselspital, Bern University Hospital, University of Bern, Bern, Switzerland; 7 Department of Radiology and Nuclear Medicine, Lucerne Cantonal Hospital, University of Lucerne, Lucerne, Switzerland

**Keywords:** Computed tomography, Portal hypertension, Noninvasive, Liver disease, Prognosis

## Abstract

**Purpose:**

This study aimed to analyze the predictive value of caudate to right lobe ratio (CRL-R) and liver segmental volume ratio (LSVR) for chronic liver disease (CLD) on routine abdominal CT scans and their association with 5-year decompensation- and transplant-free survival.

**Method:**

This retrospective study included 108 patients without CLD and 98 patients with biopsy-proven CLD. All patients underwent abdominal CT scans between 03/2015 and 08/2017. Patients with CLD were divided into three groups: early CLD (F0-F2; eCLD; n = 40), advanced CLD (F3-F4; aCLD; n = 20), and aCLD with clinically significant portal hypertension (aCLDPH; n = 38). CRL-R and LSVR were compared between groups using Kruskal–Wallis test and ROC analysis to determine cutoff-values. 5-year decompensation- and transplant-free survival were assessed by Kaplan–Meier curve analysis.

**Results:**

CRL-R and LSVR were significantly different between all groups (p < 0.001). A CRL-R cutoff-value of > 0.99 predicted aCLD with a sensitivity of 69% and a specificity of 80% (AUC = 0.75, p < 0.001), while LSVR > 0.37 had a sensitivity of 67% and a specificity of 84% (AUC = 0.80, p < 0.001). CLD-patients with both CRL-R > 0.99 and LSVR > 0.37 had a significantly lower probability of 5-year decompensation-free survival (31%) as well as lower probability of 5-year transplant-free survival (41%) than those with a CRL-R < 0.99 and/or LSVR < 0.37 (70%, 62%, p = 0.006, p = 0.038).

**Conclusion:**

CRL-R and LSVR showed a high predictive value for CLD on routine abdominal CT scans. In patients with CLD, both CRL-R and LSVR may be combined and are associated with 5-year decompensation-free and transplant-free survival.

## Introduction

Chronic liver disease (CLD) is characterized by persistent liver damage leading to liver fibrosis and cirrhosis [1]. Complications of liver cirrhosis include liver decompensation with variceal bleeding, hepatic encephalopathy, ascites, renal dysfunction, development of hepatocellular carcinoma and death [2–5]. Chronic liver disease is responsible for approximately 2 million deaths worldwide per year, mostly related to direct complications of cirrhosis and hepatocellular carcinoma [6, 7]. In western and developed countries, alcohol related liver disease (ARLD) and metabolic dysfunction-associated steatotic liver disease (MASLD) are the most common causes of chronic liver disease, while in Asia, hepatitis B is responsible for the majority of chronic liver disease cases [6]. Globally, the incidence of hepatitis B is currently decreasing [7]. Conversely, the global incidence of ARLD is increasing, particularly among young people and women [7]. In addition, the prevalence of MASLD is increasing worldwide due to the demographic trend toward an aging population and the rising prevalence of obesity and other risk factors for metabolic syndrome [8–10]. With this global increase in CLD prevalence, there is a need for simple and widely available noninvasive tools to screen for liver fibrosis and to assess the risk for future liver decompensation or liver related death on routinely performed standard abdominal computed tomography (CT) scans.

Since CLD is associated with atrophy of the right lobe and hypertrophy of the left and caudate lobe [11], quantitative measurements of liver segmental diameter and volumes are promising noninvasive methods to assess for CLD [12]. The caudate to right lobe ratio (CRL-R) represents a fast and easy measurement, that may be performed on any abdominal CT scan. CRL-R is calculated by dividing the diameter of the caudate lobe by the diameter of the right liver lobe. The initially proposed CRL-R was modified, so that the lateral wall of the main portal vein is now used as the landmark to calculate the CRL-R, and not the lateral border of the caudate lobe, as initially proposed [13, 14]. CRL-R has shown to be a helpful parameter to detect significant liver fibrosis [15]. More time consuming, the liver segmental volume ratio (LSVR) is calculated by dividing the volumes of liver segments I-III by volumes of liver segments IV-VIII. Similarly to CRL-R, LSVR has been shown to be a useful parameter for liver fibrosis staging [16].

Studies have shown the association of an increased CRL-R and LSVR with the degree of liver fibrosis [17, 18]. However, up to our knowledge, there are no studies investigating the association of CRL-R and LSVR with decompensation-free and transplant-free survival.

We hypothesized that increased CRL-R and LSVR are useful to screen for patients with CLD on routine abdominal CT scans and predict the outcome in those with detected CLD. Therefore, this study aimed to analyze the predictive value of caudate to right lobe ratio (CRL-R) and liver segmental volume ratio (LSVR) for chronic liver disease (CLD) on routine abdominal CT and its association with 5-year decompensation- and transplant-free survival.

## Material and methods

### Study population and patient groups

**Fig. 1:**
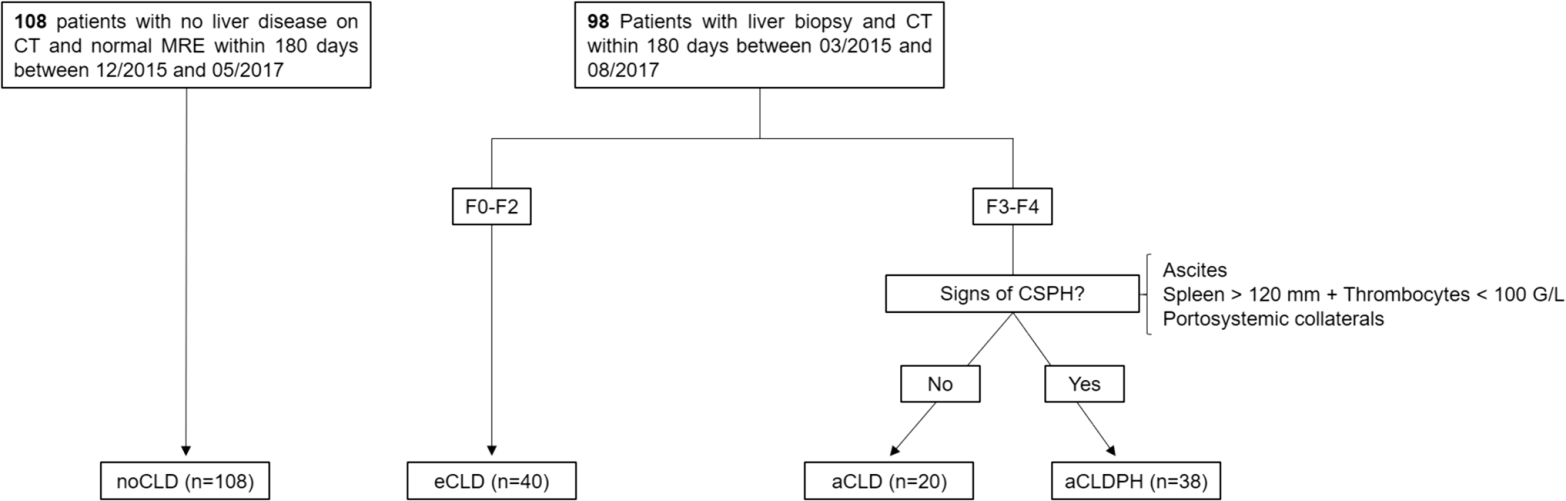
Flowchart of patient inclusion and groups. Normal MRE was defined as liver stiffness below 2.8 kPa. MRE magnetic resonance elastography, *CT* computed tomography, *CSPH* clinically significant portal hypertension, *noCLD* no chronic liver disease, *eCLD* early chronic liver disease, *aCLD* advanced chronic liver disease, *aCLDPH* advanced chronic liver disease with CSPH

The institutional review board approved this retrospective study. All recruited patients were informed and included, if they did not refuse to give their consent for study participation. A total of 206 patients were included: 98 patients with chronic liver disease without history of liver transplantation who underwent a CT scan of the abdomen and liver biopsy within 180 days between March 2015 and August 2017, as well as 108 patients without chronic liver disease who underwent a CT scan of the abdomen within 180 days of a normal magnetic resonance elastography (MRE) examination (liver stiffness < 2.8 kPa) between December 2015 and May 2017 (Fig. 1). Patients with CLD were grouped based on their fibrosis grade into early chronic liver disease (eCLD; F0-F2; n = 40), advanced chronic liver disease (aCLD; F3-F4; n = 20), and aCLD with clinically significant portal hypertension (aCLDPH; n = 38). Criteria for clinically significant portal hypertension were 1) splenomegaly with concomitant thrombocytopenia (spleen in largest axis > 120 mm + thrombocytes < 100 G/L); 2) ascites; 3) portosystemic collaterals.

### Image analysis

**Fig. 2:**
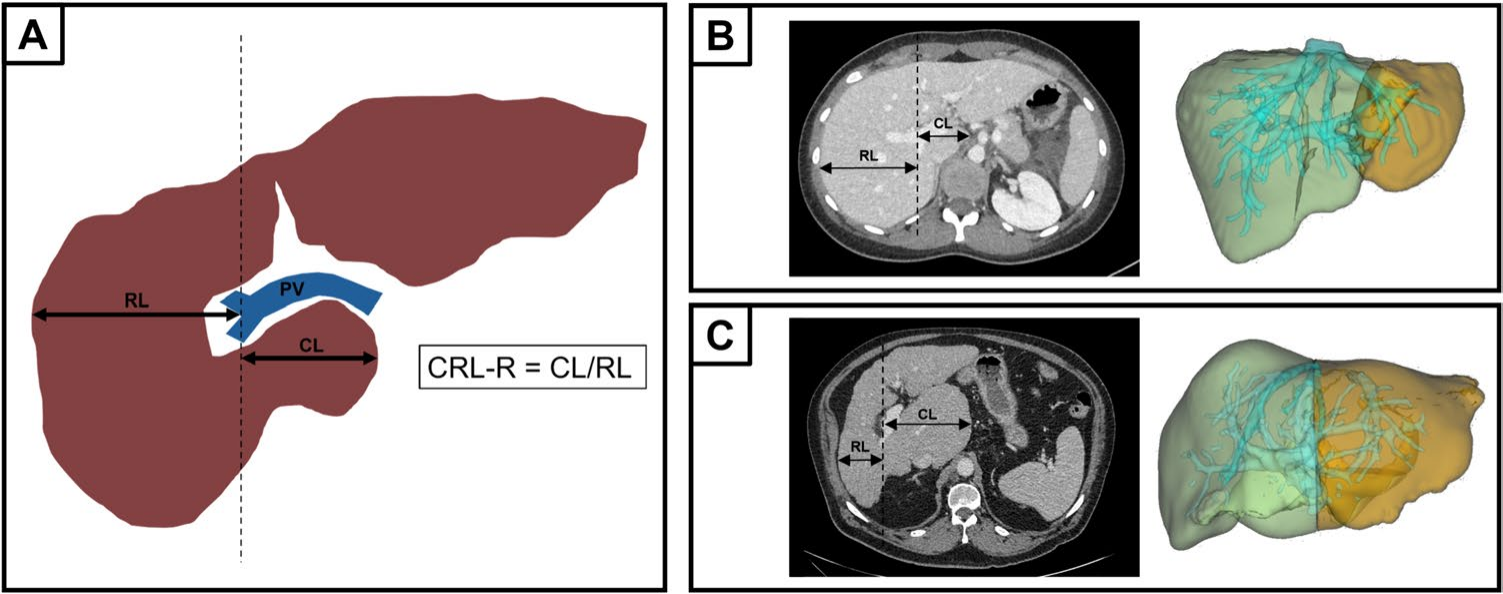
Measurement and calculation of the caudate to right lobe ratio and liver segmental volume ratio with patient examples. A) To measure the maximum diameter of the caudate lobe and right lobe, a strictly vertical straight line was first drawn through the lateral wall of the right bifurcation of the portal vein on an axial slice. This line was then used as a starting point to draw strictly horizontal straight lines to measure the maximum diameter of the caudate lobe and right lobe. B) Image examples of a 24 year old female patient without chronic liver disease. The left image shows CRL-R measurement and the right image shows semiautomatic LSVR measurement using “IntelliSpace Portal”. The LSVR was calculated by dividing the volumes of liver segments I-III (orange) by liver segments IV–VIII (light green). CRL-R was 0.62 and LSVR was 0.23 for this patient. C) Image examples of a 72 year old male patient with advanced chronic liver disease without portal hypertension. The left image shows CRL-R measurement and the right image shows semiautomatic LSVR measurement using “IntelliSpace Portal”. The LSVR was calculated by dividing the volumes of liver segments I-III (orange) by liver segments IV–VIII (light green). CRL-R was 1.32 and LSVR was 0.88 for this patient

All image analyses were performed by a single trained radiology resident (J.H.) using a venous CT phase with 1 mm slice thickness. Hereby the radiology resident was blinded to the clinical information of the patients. “IntelliSpace Portal” (Version 12.1.6, Philips Medical Systems, Veenpluis, PC Best, The Netherlands) was used for semiautomatic liver volumetry, in which the individual volumes of the liver segments (I to VIII, with IV divided into IVa and IVb) were measured. Furthermore, the clinical picture archiving software “Sectra Workstation PACS IDS7” (version 21.2, Sectra AB, Linköping, Sweden) was used to screen for signs of portal hypertension (ascites, portosystemic collaterals, splenomegaly) and to measure the maximal caudate lobe diameter and the maximal right lobe diameter. The modified CRL-R was calculated as proposed by Awaya et al. [14] and illustrated in Fig. 2. The diameter of the caudate lobe and the right lobe were measured strictly horizontal from the lateral wall of the right portal vein bifurcation to the medial border of the caudate lobe and to the lateral border of the right liver lobe. CRL-R and LSVR were then calculated using the following formulas [15, 18]:

$$CRL-R= \frac{Diameter \, of \,caudate\, lobe}{Diameter \, of \, right\, lobe}$$

$$LSVR= \frac{Volume\, segments\, I-III}{Volume\, segments\, IV-VIII}$$

RL = right lobe; CL = caudate lobe; PV = portal vein, CRL-R = caudate to right lobe ratio.

To evaluate the interrater reliability, CRL-R and LSVR were measured again by a second author (D.C.) in 30 randomly selected patients (10 patients with noCLD, 10 patients with eCLD, 5 patients with aCLD and 5 patients with aCLDPH).

### Clinical data

Clinical data collected within a three-month period of the CT examination included the etiology of liver disease, age, sex, body mass index (BMI), history of arterial hypertension, smoking, regular alcohol consumption (defined as ≥ 2 alcoholic beverages per day for men and ≥ 1 alcoholic beverage per day for women or the presence of a history of abusive alcohol consumption), diabetes mellitus, and dyslipidemia. Laboratory parameters collected within the same timeframe included creatinine, albumin, bilirubin, aspartate aminotransferase (AST), alanine aminotransferase (ALT), alkaline phosphatase (ALP), gamma-glutamyltransferase (GGT), low density lipoprotein (LDL), triglycerides, thrombocytes and international normalized ratio (INR). A 5-year follow-up was conducted for each patient, documenting orthotopic liver transplantation (OLT) or all-cause death. For patients without any prior decompensation at the time of the CT scan, first hepatic decompensation during a 5-year follow-up was noted (variceal hemorrhage, jaundice, hepatorenal syndrome, hepatic encephalopathy).

### Statistical analyses

Statistical analyses were performed using GraphPad Prism (version 10.0.2, GraphPad Software, San Diego, California, USA) and IBM SPSS Statistics (version 25.0, IBM Corporation, Armonk, New York, USA). Nonparametric tests were used for all analyses. Parameters among noCLD, eCLD, aCLD and aCLDPH were compared using the Kruskal–Wallis test with Dunn’s multiple comparison post hoc test for continuous variables or the χ2-test with a post hoc test consisting of a Z-test with Bonferroni correction for categorical variables [19]. Receiver operating characteristic (ROC) analyses for group differentiation were performed for CRL-R and LSVR and cutoff values were determined using Youden’s index. In addition, ROC analyses for a combination of CRL-R and LSVR were generated using a multiple logistic regression analysis. Furthermore, Kaplan–Meier curves over a 5-year period were generated for single parameters (CRL-R, LVSR) and combined parameters (CRL-R + LSVR) using the optimal cutoff values for detection of patients with advanced CLD, as obtained using the ROC analyses.

The Kaplan–Meier curves analyzed the following two endpoints: 1) Decompensation-free survival. First hepatic decompensation was defined as the first occurrence of variceal hemorrhage, jaundice, hepatorenal syndrome, or hepatic encephalopathy in patients without prior hepatic decompensation at the time of CT. 2) Transplant-free survival, whereas the occurrence of liver transplant or death was defined as a composite endpoint. Both endpoints were assessed in all patients, as well as in patients with CLD.

The interrater reliability for CRL-R measurement and LSVR measurement was assessed by calculating an intraclass correlation coefficient (ICC) for each measurement using a two-way mixed effect model with an ICC definition of absolute agreement [20]. Hereby, an ICC below 0.5 was defined as poor reliability, between 0.5 and 0.75 as moderate reliability, between 0.75 and 0.9 as good reliability and above 0.9 as excellent reliability [20].

For all statistical tests, a p-value of 0.05 or less was defined as significant.

## Results

### Patient characteristics

Patient characteristics are shown in Table 1. Patients with CLD were significantly older, showed a higher prevalence of regular alcohol consumption, diabetes mellitus and dyslipidemia than patients without CLD. Not surprisingly, patients with CLD had higher liver enzyme tests, as well as lower albumin, thrombocyte count and higher INR values. The most common etiology of CLD was alcohol related liver disease (22%), followed by metabolic dysfunction-associated steatotic liver disease (17%), chronic viral hepatitis (17%), autoimmune hepatitis (12%), drug induced liver injury (6%), cholestatic liver disease (5%), and mixed etiologies (16%).

**Table 1:** Patient characteristics

Parameter	noCLD (n = 108)	eCLD (n = 40)	aCLD (n = 20)	aCLDPH (n = 38)	p-value
Age (years)	52 (41–61)	56 (41–69)	63 (57–69)*	61 (53–70)*	< 0.001
Male (n, %)	49 (45)	20 (50)	11 (55)	25 (66)	0.184
BMI (kg/m^2^)	25 (23–29)	23 (21–30)	27 (24–31)	28 (23–31)	0.193
Arterial hypertension (n, %)	22 (20)	16 (40)	9 (45)	14 (37)	0.021
Smoking (n, %)	18 (17)	7 (18)	5 (25)	8 (21)	0.803
Regular alcohol consumption (n, %)	0 (0)	4 (10)*	7 (35)*	18 (47)*/**	< 0.001
Diabetes mellitus (n, %)	5 (5)	8 (20)*	5 (25)*	10 (26)*	0.001
Dyslipidemia (n, %)	8 (7)	14 (35)*	6 (30)*	10 (26)*	< 0.001
Creatinine (μmol/L)	76 (65–90)	73 (62–97)	74 (63–97)	74 (65–93)	0.996
Albumin (g/L)	37 (35–39)	30 (20–36)*	34 (26–36)*	32 (26–36)*	< 0.001
Bilirubin (μmol/L)	7 (5–13)	13 (9–64)*	16 (11–26)*	21 (12–28)*	< 0.001
AST (U/L)	22 (19–26)	40 (31–143)*	74 (52–114)*	51 (34–83)*	< 0.001
ALT (U/L)	22 (17–34)	59 (34–207)*	69 (49–106)*	39 (21–54)***	< 0.001
ALP (U/L)	72 (53–84)	143 (109–189)*	157 (82–219)*	111 (75–157)*	< 0.001
GGT (U/L)	21 (16–38)	171 (99–302)*	315 (106–379)*	124 (63–244)*	< 0.001
LDL (mmol/L)	2.5 (2.0–3.0)	3.3 (2.7–4.0)	3.0 (1.9–3.7)	2.2 (1.9–3.1)**	0.018
Triglycerides (mmol/L)	1.7 (1.0–2.2)	1.2 (1.0–1.8)	1.2 (1.2–1.4)	1.1 (0.9–1.3)	0.086
Thrombocytes (G/L)	224 (191–277)	221 (110–240)	202 (160–276)	125 (87–160)*/**/***	< 0.001
INR	1.0 (1.0–1.0)	1.1 (1.0–1.2)*	1.1 (1.0–1.3)*	1.1 (1.0–1.2)*	< 0.001

*noCLD* no chronic liver disease, *eCLD* early chronic liver disease, *aCLD* advanced chronic liver disease, *aCLDPH* advanced chronic liver disease with CSPH, *BMI* body mass index, *AST* aspartate aminotransferase, *ALT* alanine aminotransferase, *ALP* alkaline phosphatase, *GGT* gamma-glutamyltransferase, *LDL* low densitiy lipoprotein, *INR* International Normalized Ratio

Results are presented as median and interquartile range (25–75%). P-values were calculated using the Kruskal–Wallis test with Dunn’s multiple comparison post hoc test or χ2-test with a post hoc test consisting of a Z-test with Bonferroni correction as appropriate. * = p < 0.05 in post hoc test with noCLD; ** = p < 0.05 in post hoc test with eCLD; *** = p < 0.05 in post hoc test with aCLD. Regular alcohol consumption was defined as ≥ 2 alcoholic beverages per day for men and ≥ 1 alcoholic beverage per day for women or history of abusive alcohol consumption

### Comparison of CRL-R and LSVR between groups

CRL-R showed a good discriminative value to separate between noCLD and CLD and was the only parameter to differentiate significantly between noCLD and eCLD (Table 2). In comparison to CRL-R, LSVR showed a better discrimination between patients with eCLD and aCLD (Table 2). However, none of the analyzed parameters differed significantly between patients with and without portal hypertension (Table 2).

**Table 2:** Results of liver measurements

	noCLD (n = 108)	eCLD (n = 40)	aCLD (n = 20)	aCLDPH (n = 38)	p-value
CRL-R	0.85 (0.79–0.93)	0.97 (0.89–1.06)*	1.05 (0.95–1.29)*	1.02 (0.87–1.17)*	< 0.001
LSVR	0.27 (0.22–0.31)	0.28 (0.21–0.36)	0.43 (0.31–0.55)*/**	0.41 (0.30–0.59)*/**	< 0.001

*noCLD* no chronic liver disease, *eCLD* early chronic liver disease, *aCLD* advanced chronic liver disease, *aCLDPH* advanced chronic liver disease with CSPH, *CRL*-*R* caudate to right lobe ratio, *LSVR* liver segmental volume ratio

Parameter values for each group are presented as median and interquartile range (25–75%). P-values were calculated using the Kruskal–Wallis test with Dunn’s multiple comparison post hoc test. * = p < 0.05 in Dunn’s multiple comparison test in comparison with noCLD, ** = p < 0.05 in Dunn’s multiple comparison test in comparison with eCLD

### ROC analysis

Those observations were confirmed in the ROC analysis, showing a high area under the curve (AUC) for CRL-R to differentiate between noCLD and CLD (AUC 0.78, p < 0.001; Table 3). With a cutoff value of > 0.93, a sensitivity of 69% and a specificity of 78% for CLD was achieved. A combination of CRL-R with LSVR led to a slightly higher AUC of 0.83 with a positive predictive value (PPV) for CLD of 0.85 and a negative predictive value (NPV) of 0.64.

**Table 3:** ROC results

Parameters and cutoff-values	AUC	Sensitivity, %	Specificity, %	NPV	PPV	Youden’s index	p value
noCLD vs. CLD							
CRL-R (> 0.93)	0.78	69	78	0.74	0.74	47	< 0.001
LSVR (> 0.35)	0.72	52	85	0.66	0.76	37	< 0.001
CRL-R (> 0.93) + LSVR (> 0.35)	0.83	41	94	0.64	0.85	35	< 0.001
noCLD or early CLD vs. advanced CLD							
CRL-R (> 0.99)	0.75	69	80	0.87	0.57	49	< 0.001
LSVR (> 0.37)	0.80	67	84	0.86	0.63	51	< 0.001
CRL-R (> 0.99) + LSVR (> 0.37)	0.85	52	96	0.84	0.86	48	< 0.001
Patients without PH vs. patients with PH							
CRL-R (> 0.99)	0.69	68	74	0.91	0.37	42	< 0.001
LSVR (> 0.37)	0.76	68	80	0.92	0.43	48	< 0.001
CRL-R (> 0.99) + LSVR (> 0.37)	0.79	53	91	0.89	0.57	44	< 0.001

*ROC* receiver operating characteristic, *AUC* area under the curve, *NPV* negative predictive value, *PPV* positive predictive value, *noCLD* no chronic liver disease, *CLD* chronic liver disease, *PH* portal hypertension, *CRL*-*R* caudate to right lobe ratio, *LSVR* liver segmental volume ratio

For the detection of patients with advanced CLD, LSVR (AUC 0.80; p < 0.001) showed a better performance than CRL-R (AUC 0.75; p < 0.001). A LSVR cutoff value of > 0.37 showed a sensitivity of 67% and a specificity of 84%, for detection of aCLD. Therefore, a LSVR below 0.37 was able to rule out advanced CLD with a NPV of 0.86. A combination of LSVR with CRL-R increased the AUC to 0.85 with a NPV of 0.84 (p < 0.001).

Prediction of clinically significant portal hypertension (CSPH) could be achieved with LSVR (cutoff-value 0.37, AUC 0.76, p < 0.001, sensitivity of 68%, specificity of 80%). A combination of CRL-R with LSVR just slightly increased the performance (AUC of 0.79; p < 0.001).

### Relationship between liver segmental volume parameters and 5-year survival in all patients (with and without chronic liver disease)

**Fig. 3:**
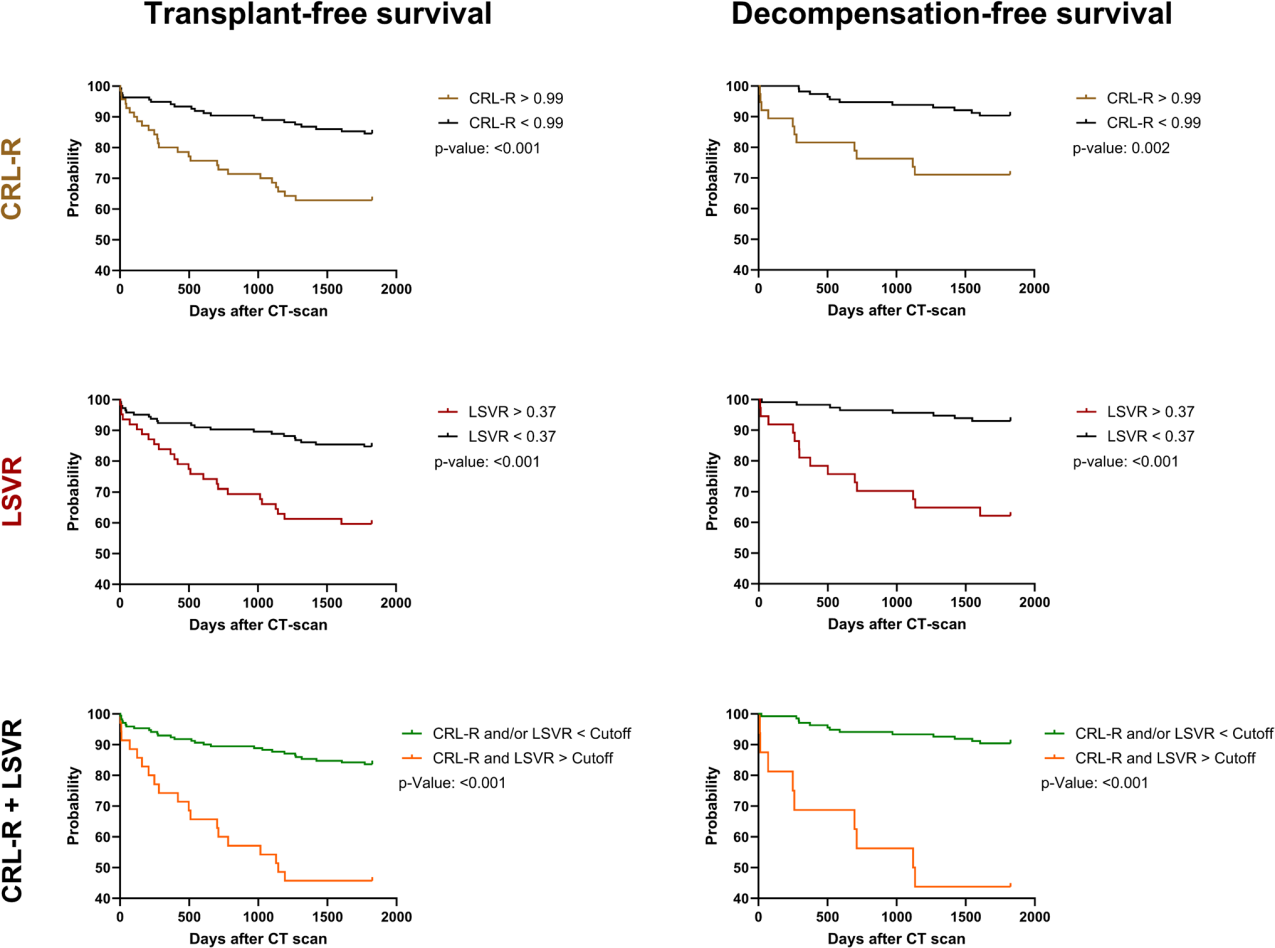
Kaplan–Meier curves of all patients (noCLD and CLD). *CRL*-*R* caudate to right lobe ratio, *LSVR* liver segmental volume ratio

Patients with a CRL-R above 0.99 had a significantly lower probability of 5-year transplant-free survival (63%), compared to patients with a CRL-R below 0.99 (85%, p < 0.001). Similarly, an LSVR above 0.37 was associated with a lower probability of 5-year transplant-free survival (60%), compared to patients with an LSVR below 0.37 (85%, p < 0.001). Patients with both CRL-R > 0.99 and LSVR > 0.37 showed the lowest probability of 5-year transplant-free survival (46%), which was significantly different (p < 0.001) from CLD-patients with CRL-R < 0.99 and/or LSVR < 0.37 (84%) (Fig. 3).

Similar observations could be made for the 5-year decompensation-free survival, whereby patients with a CRL-R above 0.99 were significantly less likely to have a decompensation-free 5-year survival (72%) than patients with a CRL-R below 0.99 (90%, p = 0.002). Furthermore patients with a LSVR above 0.37 were significantly less likely to have a decompensation-free 5-year survival (62%) compared to patients with an LSVR below 0.37 (93%, p < 0.001). Patients with both CRL-R > 0.99 and LSVR > 0.37 showed the lowest probability of 5-year decompensation-free survival (44%), which was significantly different (p < 0.001) from patients with CRL-R < 0.99 and/or LSVR < 0.37 (90%) (Fig. 3).

### Relationship between liver segmental volume parameters and 5-year survival only in patients with chronic liver disease

**Fig. 4:**
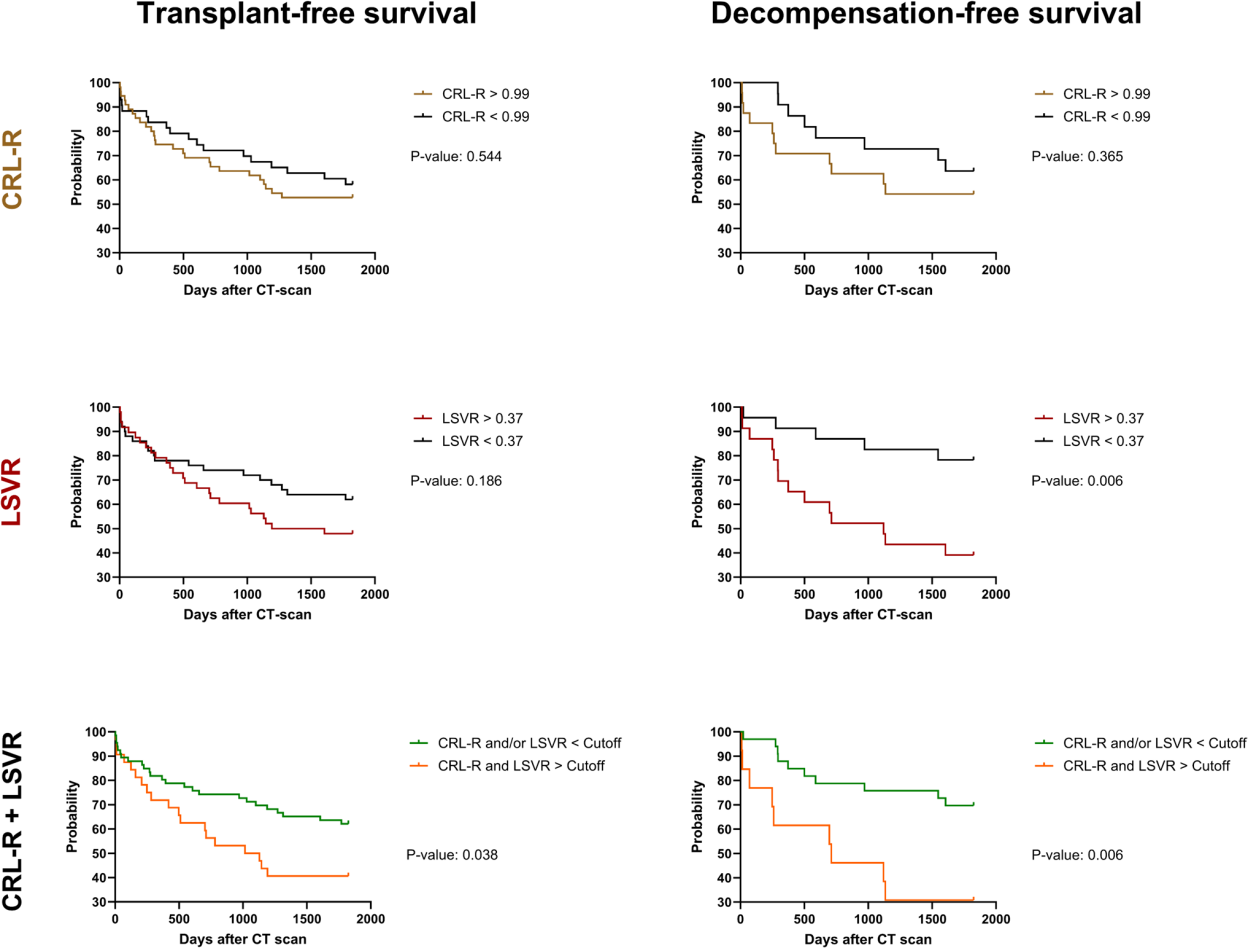
Kaplan–Meier curves of subanalysis with only CLD-patients. *CRL*-*R* caudate to right lobe ratio, *LSVR* liver segmental volume ratio

Among the three models proposed for predicting the 5-year transplant-free survival in patients with chronic liver disease, only the combination of CRL-R and LSVR demonstrated significant outcomes. Patients with both a CRL-R above 0.99 and a LSVR above 0.37 exhibited a lower likelihood of 5-year transplant-free survival (41%), in contrast to patients with a CRL-R and/or a LSVR above the specified cutoff (62%, p = 0.038) (Fig. 4).

Regarding the risk-stratification of 5-year decompensation-free survival in patients with chronic liver disease, only LSVR and a combination of CRL-R and LSVR achieved to help predict the 5-year decompensation-free survival in patients with CLD. Hereby, patients with a LSVR above 0.37 showed a lower likelihood of 5-year decompensation-free survival (39%) when compared to patients with a LSVR below 0.37 (78%, p = 0.006). In a combination of CRL-R and LSVR, patients with both CRL-R above 0.99 and LSVR above 0.37 showed a much lower 5-year decompensation-free survival-rate (31%) compared to patients with CRL-R < 0.99 and/or LSVR < 0.37 (70%, p = 0.006) (Fig. 4).

### Interrater reliability

Good interrater reliability (ICC of 0.88) was observed for the CRL-R measurement, while excellent interrater reliability (ICC of 0.99) was observed for the LSVR measurement.

## Discussion

This study demonstrates that CRL-R and LSVR are useful noninvasive parameters to screen for liver fibrosis on routine abdominal CT scans and to predict the 5-year probability of decompensation- and transplant-free survival. In patients with unknown CLD, CRL-R is the fastest and most accurate parameter to rule out chronic liver disease and predict the transplant-free survival rate and the hepatic decompensation risk over a period of 5 years. With a CRL-R cutoff value of > 0.93 patients with CLD could be differentiated from patients without CLD with a sensitivity of 69% and a specificity of 78%. In patients with an increased CRL-R, an additional LSVR > 0.37 led to a specificity to 96% with a PPV of 0.86. A practical approach in a routine abdominal scan to detect patients with CLD would therefore be to measure the modified CRL-R in all patients and calculate LSVR in those with a CRL-R above 0.93. Among those, a LSVR above 0.37 is associated with a worse prognosis.

In patients with known CLD, a combination of CRL-R > 0.99 and LSVR > 0.37 showed the lowest probability of 5-year transplant-free survival (41%), which was significantly different (p = 0.038) from patients with CRL-R < 0.99 and/or LSVR < 0.37 (62%). Similarly, patients with known CLD and both CRL-R above 0.99 and LSVR above 0.37 showed a much lower 5-year decompensation-free survival-rate (31%) compared to patients with CRL-R < 0.99 and/or LSVR < 0.37 (70%) (p = 0.006). CRL-R and especially LSVR may therefore be promising noninvasive imaging biomarkers for risk stratifications in patients with known CLD and should be investigated in a larger cohort of patients with advanced CLD with and without portal hypertension.

Our results are in agreement with previously published results of other authors. CRL-R and LSVR are known to be higher in patients with liver fibrosis and cirrhosis, respectively, than in patients without liver fibrosis [14, 15, 18, 21, 22]. Also, LSVR is known to be increased in patients with CSPH [23]. However, to our knowledge, there are no published studies on CRL-R in the context of CSPH. Furthermore, to our knowledge, no previous studies investigated the prognostic benefit of CRL-R and LSVR in the context of chronic liver diseases. Based on the results of the present study, calculation of LSVR in patients with increased CRL-R is highly predictive for 5-year liver decompensation and death.

Apart from CRL-R and LSVR, there are other CT parameters that have been used for noninvasive diagnosis of fibrosis and portal hypertension, such as the liver surface nodularity (LSN) [24]. Several studies have shown that the liver surface nodularity score increases with the degree of fibrosis [25–28]. It has also been shown that patients with CSPH have a higher LSN score than patients without CSPH [29]. Similarly, from the perspective of magnetic resonance imaging, some parameters have recently been successfully used to estimate the degree of fibrosis of the liver and portal hypertension, including T1 mapping techniques. T1 maps before and after contrast administration can be used to calculate a T1 reduction rate. The T1 reduction rate is known to correlate significantly with the degree of liver fibrosis [30]. In addition, it has been shown that the T1 reduction rate can be helpful in the detection of clinically significant portal hypertension on routine liver MRI [31]. Consequently, it is worth considering that a combination of CRL-R, LSVR and multiparametric MRI parameters such as T1 mapping may be used to determine the degree of fibrosis even more accurately and to detect portal hypertension even better than each parameter alone, however this warrants further clarification.

This study has several limitations. First, there was a retrospective study design. However, thanks to stringent inclusion criteria and correlation with liver biopsy and MR elastography, the patient population was well characterized. A further limitation of this study is the relatively small number of patients with advanced chronic liver disease, which precludes any definitive conclusions regarding the significance of CRL-R and LSVR with regard to disease progression in this specific patient group. Consequently, dedicated future studies on CRL-R and LSVR as predictors of disease progression in patients with advanced chronic liver disease are warranted.

A strength of the present study is that all patients with chronic liver disease had a detailed and well-documented 5-year follow-up. Another potential limitation of this study is the lack of invasive measurement of the hepatovenous pressure gradient. However, based on liver histology and clinically relevant criteria, clinically significant portal hypertension could be determined in a large population in a realistic clinical setting.

## Conclusion

CRL-R and LSVR showed a high predictive value for CLD on routine abdominal CT scans. In patients with CLD, both CRL-R and LSVR may be combined and are associated with 5-year decompensation-free and transplant-free survival.

## Data Availability

No datasets were generated or analysed during the current study.

## Abbreviations

aCLD: Advanced chronic liver disease
aCLDPH: Advanced chronic liver disease with clinically significant portal hypertension
ALP: Alkaline phosphatase
ALT: Alanine aminotransferase
ARLD: Alcohol related liver disease
AST: Aspartate aminotransferase
AUC: Area under the curve
BMI: Body mass index
CLD: Chronic liver disease
CRL-R: Caudate to right lobe ratio
CSPH: Clinically significant portal hypertension
CT: Computed tomography
eCLD: Early chronic liver disease
GGT: Gamma-glutamyltransferase
HVPG: Hepatovenous pressure gradient
ICC: Intraclass correlation coefficient
INR: International Normalized Ratio
LDL: Low density lipoprotein
LSN: Liver surface nodularity
LSVR: Liver segmental volume ratio
MASLD: Metabolic dysfunction-associated steatotic liver disease
MRE: Magnetic resonance elastography
noCLD: No chronic liver disease
NPV: Negative predictive value
OLT: Orthotopic liver transplantation
PPV: Positive predictive value
ROC: Receiver operating characteristic
